# Investigation of ortho-positronium annihilation for porous materials with different geometries and topologies

**DOI:** 10.1038/s41598-023-40901-3

**Published:** 2023-08-22

**Authors:** Nguyen Thanh Trung, Nguyen Thuy Duong, Nguyen Quoc Hien, Tran Duy Tap, Nguyen Duc Thanh

**Affiliations:** 1 Institute of Physics, Vietnam Academy of Science and Technology, Ba Dinh, Hanoi, Vietnam; 2 Vietnam Japan University, Vietnam National University, Hanoi, Vietnam; 3 Vietnam Atomic Energy Institute, 59 Ly Thuong Kiet, Hanoi, Vietnam; 4 Faculty of Materials Science and Technology, University of Science, Vietnam National University, Ho Chi Minh City, Vietnam; 5 Research and Development Center for Advanced Technology, Cau Giay, Hanoi, Vietnam

**Keywords:** Chemistry, Materials science, Physics

## Abstract

In this work, we present the results of the ortho-positronium (o-Ps) annihilation lifetimes and nitrogen adsorption measurements for different porous materials and an approach for describing the annihilation of o-Ps in a pore, which results in a surface-volume formula (SVF) for calculating the pore-related o-Ps lifetime. This proposed formula gives the relationship between the o-Ps annihilation rate and the effective pore radius, bulk composition, and pore structure, including pore geometry and topology. The pore-related o-Ps lifetimes of different materials calculated by the SVF are consistent with experimental results for both micro- and mesopores (and macropores) with different geometries and topologies. The SVF is convenient for calculations of pore dimensions for many cases of metal organic frameworks and zeolites. This approach enables us to fully explain the temperature dependence of the o-Ps annihilation lifetime over a wide temperature range, 20–700 K.

## Introduction

Nanoporous materials have been intensively researched for synthesis and applications because of their potential applications in adsorption, hydrogen storage, catalysis, bioseparation, gas separation, and energy storage^1–4^. These characteristics are influenced by the pore morphology, topology, geometrical size, surface area, and surface-to-volume ratio S/V^5–8^ (S and V are the pore surface area and volume, respectively), which are directly related to the reaction rate of catalysts, diffusion of small molecules, heat conduction, thermoregulation of objects, and selective absorption of molecules^1–8^. Conventionally, gas adsorption measurements^9^ have been used for the determination of pore surface area and volume; however, this approach presents challenges when working with microporous materials^9^, closed pores and thin films^10^. In addition to the diffraction method and density functional calculations^11^, positron annihilation lifetime spectroscopy (PALS) is a powerful method used for characterizing the pore structures of nanoporous materials^10,12,13^. Positronium is a metastable bound state of a positron and an electron, formed in bulk-sized insulators or at the pore surface, which can be used to elucidate pore structure in such materials. Positronium can be formed as a singlet state, ^1^S_0_, with antiparallel spins (S = 0), known as para-positronium (p-Ps), or it can be formed as a triplet state, ^3^S_1_, with parallel spins (S = 1), known as ortho-positronium. The p-Ps state has a short lifetime of 125 ps, while o-Ps has a longer lifetime of 142 ns in vacuum. In the medium of materials, the lifetime of o-Ps is shortened due to the pick-off annihilation with surrounding electrons. In porous media, the o-Ps lifetimes are reported to be closely correlated with the pore radius^12,13^.

There are several models related to o-Ps annihilation in pores^12–21^. Tao^12^ modelled small pores by a finite potential well with a finite thickness of the electron cloud, ΔR, in the bulk surrounding the pore, in which o-Ps undergoes pick-off annihilation with a constant rate of 2 ns^−1^. The probability of o-Ps penetrating in this layer is reported to be proportional to the pore size^12^. For convenience of calculation, Eldrup et al.^13^ later calculated the pore-related o-Ps lifetime using the normalized wavefunction of the o-Ps in an infinite potential well and proposed the widely used formula named the Tao-Eldrup (TE) model^12,13^, which has been successfully applied for subnanometre-sized spherical micropores. However, this model failed to predict the experimental results for pores with radii exceeding 1 nm^14^. Consolati et al*.*^16^ extended the TE model for larger pores with different geometrical considerations. Another way to extend the TE model can be found in the works of Goworek et al.^17^ and Ciesielski et al.^18^, who were the first to propose integrating all the populations of possible excited states of a particle in a spherical (or cylindrical) infinite potential well. However, due to mathematical difficulties, Goworek et al.^17^ and Ciesielski et al.^18^ were unable to provide a quantitative formula for the relationship between pore size and mean o-Ps annihilation rate. Gidley et al.^19^ and Dull et al*.*^20^ developed this approach and successfully integrated the populations of the thermally excited o-Ps states derived from the Schrödinger equation using a rectangular infinite potential. This led to the TE-extended rectangular (RTE) model^19,20^. The main features of the above extensions are using the normalized wavefunctions of the o-Ps state in an infinite potential well to calculate the probability of o-Ps in the electron layer, ΔR, inside an expanded pore^13,16–20^. The pick-off annihilation rate is assumed to be a constant of 2 ns^−1^ in this electron layer, ΔR, for all materials^12–20^. Using the Boltzmann distribution for thermal excited states of the o-Ps, the RTE formula can explain the dependence of the o-Ps lifetime on temperatures higher than 273 K. The above RTE extensions have gained an advantage in extending the calculations of the o-Ps lifetimes for pore sizes larger than that of the TE model and explaining the o-Ps lifetime dependence in the high-temperature range. However, these solutions are still incomplete. The applications of such models for many cases of mesopores show disagreements with experimental results. The temperature dependence of the o-Ps lifetime calculated by the RTE model is inconsistent with experimental results^21^ at temperatures lower than 273 K. It is the fact that using an infinite potential well is convenient for the analytical derivation of the Schrödinger equation; however, it is not appropriate for larger pore sizes. In addition to the above solution for extending the TE model for large pores, the factor κ, explained as the relative contact density^16^, can be introduced^16^ for the correction of 3γ-intrinsic annihilation. However, the analytical expression of the pore radius dependence of this correction factor, κ, has not been found. Another way to extend the TE model can be found in the work of Ito et al.^14^. Instead of adjusting the factor κ for the self-decaying rate in the medium, Ito et al.^14^ adjusted the fraction of the o-Ps probability related to the pick-off annihilation by multiplying that of the TE formula^13^ by an empirical decreasing function of the pore radius. This practice solution has successfully calculated the o-Ps annihilation lifetime for larger pores^14^. Nevertheless, the formula of Ito et al*.*^14^ and simple model of Wada et al.^15^ cannot deal with pores with different shapes, which strongly influence the o-Ps lifetime calculations, and it did not take into account the explanation for the temperature dependence of the o-Ps lifetime. Furthermore, the influences of bulk composition^22^ and pore topology^23^ have not been considered by the aforementioned works^12–21^. To extend the TE model for complex geometries of the pore network, Zubia et al.^23^ recently implemented the atomic model and provided a numerically calculated result for microporous zeolites. However, to better interpret the o-Ps lifetimes associated with various pore sizes and topologies of different types of porous materials, the aforementioned models^12–20,23^ are suggested to be improved by another approach that is different from those of TE-extended models. Again, regarding the reason for the limitation of the RTE model in explaining the o-Ps lifetime-dependent on the low temperature range, it is suggested that the use of the Boltzmann distribution for the population of o-Ps states is suitable only for the high temperature range but is not appropriate for low temperatures. The dependence of the o-Ps lifetime on the low temperature range has been explained later by Dutta et al.^21^ Nevertheless, the empirical formula of Dutta et al.^21^ is unsuitable for temperatures greater than 273 K. These problems have not been completely resolved by existing models^12–21,23^. Meanwhile, research studies using PALS have been increasing. Researchers are interested in applying the PALS method to various porous materials, such as MOFs^24,25^, thin films^10,26,27^, microporous zeolites^28,29^ and polymers^30^, mesoporous silica^31^ and zeolites^32^.

To consider the characteristics of o-Ps annihilation associated with different porous materials of different pore sizes, bulk compositions, pore geometries and topologies, in this work, we present experiments with the results of both PALS and nitrogen adsorption–desorption measurements for different porous materials and an approach using a finite potential well for o-Ps annihilation in a pore. Relating to the model of o-Ps annihilation in a pore, we assume that o-Ps undergoes pick-off annihilation in both regions, including the outer^12,13^ and inner^21^ electrons of the pore volumes. Here, the electron density in the pore inner volume is assumed to arise due to the thermal atomic vibration amplitude at the pore surface^21,33^, and the electron density in the pore outer volume is considered to be the bulk electron density^12,16–20^. This approach has resulted in the surface-volume formula (SVF) giving the o-Ps calculation of the o-Ps annihilation rate depending on the surface-to-volume ratio and on the temperature. The effect of bulk composition on the o-Ps lifetime is included. SVF is suitable for calculations of both micro- and mesopores. Experimentally, we perform PALS measurements for the MOF of AL-Mil-53^34^, zeolites ZSM-5^2,35^, MCM-41^36^, zeolites 5A^37^ and 13X^38^. The experimental results of AL-Mil-53, ZSM-5 and MCM-41 are also used to calibrate the SVF calculations into experimental results for the determination of the SVF parameters by a consistent system of transcendental equations. Notably, this procedure is needed and found in many other models^13,14,19,20^ in which the RTE calculations are calibrated by using the TE calculations^13^. Al-Mil-53 is a metal–organic framework with a Mil-53 pore topology and a large breathing effect^34^, ZSM-5^35^ is a hierarchical microporous zeolite with *mfi* topology, and MCM-41 is a mesoporous silica material with a channel shape. These materials are currently receiving much attention due to their unique properties in gas adsorption, removal of chemical and biological compounds, catalysis, and industrial use^2,37,39–43^. We did not carry out gas adsorption measurements for 5A and 13X to calibrate the SVF calculation with the PALS results. However, the measured o-Ps lifetimes associated with 5A and 13X can be used to calculate their pore sizes, which is consistent with other reports. The o-Ps lifetimes calculated by SVF for micropores approximately agree with those calculated by the TE and RTE models (pore diameters < 2 nm) and agree with other experimental data in the literature obtained from micropores and mesopores. The temperature dependence of the o-Ps annihilation simulated by the SVF is consistent with the experimental results of the literature^18–21^ in the range of 20–700 K. The experimental results of the o-Ps lifetimes of this work and related references are performed under vacuum conditions.

## Results

### The experimental results

The experimental results are presented in Tables 1 and 2. The o-Ps lifetimes of all samples, AL-Mil-53, ZSM-5, MCM-41, zeolites 5A and 13X, are shown in Table 1, in which τ_1_–τ_4_ are lifetime components with intensities I_1_–I_4_. The results of the surface areas and volumes of MCM-41, ZSM-5 and Al-Mil-53, which represent different pore sizes, geometries and topologies, are presented in Table 2. Here, S_BET_ is the surface area given by the Brunauer–Emmett–Teller (BET) equation^9^, S_ext_, V_ads_, S_micro_, and V_micro_ are the external surface area, adsorption volume, microsurface area, and micropore volume, respectively, obtained from isotherm adsorption measurements and the t-plot method^9,44^. Additionally, S_micro_ and V_micro_ are associated with the micropores of ZSM-5 and Al-Mil-53 and are used to calculate the ratio 3 V/S used to calibrate the SVF into experimental results. For the case of MCM-41, the measurement shows no micropores, the pore surface area and volume of mesopores are ascribed to S_BET_ and V_ads_, and the effective pore radius is calculated by 3V_ads_/S_BET_ and is also used for the calibration to calculate the parameters of the SVF expression. For zeolites 5A and 13X, we performed the measurement for positron lifetimes only but did not carry out measurements of nitrogen adsorption for these samples. This is because their narrow pores with radii of subnanometres are not quite effectively accessed by gas adsorption methos^9^. Furthermore, there may be a difference in the positronium and nitrogen adsorption on these micropores; therefore, the calibration and comparison of SVF calculations with the experimental results of nitrogen adsorption for 5A and 13X are not considered. Note that ZSM-5 and AL-Mil-53 are microporous materials with pore radii of subnanometres; however, they also contain larger pores in their network structures. The results of pore surface areas and volumes of ZSM-5 and Al-Mil-53 presented in Table 2 are suggested to be associated with the pore of the effective radii, defined as 3 V/S, greater than 1 nm. These experimental results show that the pore-related o-Ps lifetime depends not only on the pore sizes but also on the bulk composition and structure, pore geometry and topology. Namely, for the cases of ZSM-5 and Al-Mil-53, although both have the same effective pore radii, the pore-related lifetime, τ_4_, associated with ZSM-5 is greater than that of Al-Mil-53. This fact is beyond the interpretation of previous models^19–21^. Note that although ZSM-5, 5A and 13 X are microporous materials with pore radii of subnanometres, the pore geometry and topology of ZSM-5, different from those of 5A and 13X, have created larger pores with long lifetime components. The presented results clearly show that the long lifetimes of ZSM-5 and Al-Mil-53 are induced by the main pore network, not by defects.

**Table 1:** The results of PALS measurements deconvoluted by PALSfit fitting^45^.

Samples	τ_1_ (ns)	τ_2_ (ns)	τ_3_ (ns)	τ_4_ (ns)	I_1_ (%)	I_2_ (%)	I_3_ (%)	I_4_ (%)	χ^2^
MCM-41	0.184 ± 0.003	0.470 ± 0.007	2.87 ± 0.09	43.40 ± 0.56	63.4 ± 0.8	26.6 ± 0.8	2.0 ± 0.1	8.0 ± 0.1	1.032
ZSM-5	0.200 ± 0.002	0.600 ± 0.004	3.54 ± 0.09	35.80 ± 0.88	48.9 ± 0.4	42.1 ± 0.4	3.0 ± 0.5	6.0 ± 0.8	1.034
Al-Mil-53	0.209 ± 0.003	0.490 ± 0.009	3.14 ± 0.20	32.20 ± 3.42	71.0 ± 1.1	25.1 ± 1.1	1.4 ± 0.1	2.5 ± 0.2	1.030
Zeolite 5A	0.167 ± 0.003	0.530 ± 0.006	2.22 ± 0.17	5.21 ± 0.10	38.9 ± 0.6	48.7 ± 0.4	4.8 ± 0.3	7.7 ± 0.4	1.065
Zeolite 13X	0.167 ± 0.003	0.520 ± 0.006	2.73 ± 0.15	6.01 ± 0.16	39.6 ± 0.4	45.8 ± 0.5	7.0 ± 0.4	7.6 ± 0.5	1.020

**Table 2:** The results of gas adsorption–desorption analysis.

Samples	S_BET_ m^2^/g)	S_ext_ (m^2^/g)	V_ads_ (cm^3^/g)	S_micro_ (m^2^/g)	V_micro_ (cm^3^/g)
MCM-41	935 ± 18	–	0.716 ± 0.015	0	0
ZSM-5	284 ± 3	177 ± 1	0.270 ± 0.006	107 ± 3	0.056 ± 0.001
Al-Mil-53	1193 ± 25	328 ± 6	1.470 ± 0.030	865 ± 26	0.458 ± 0.003

### Modelling o-Ps annihilation in a pore

**Figure 1:**
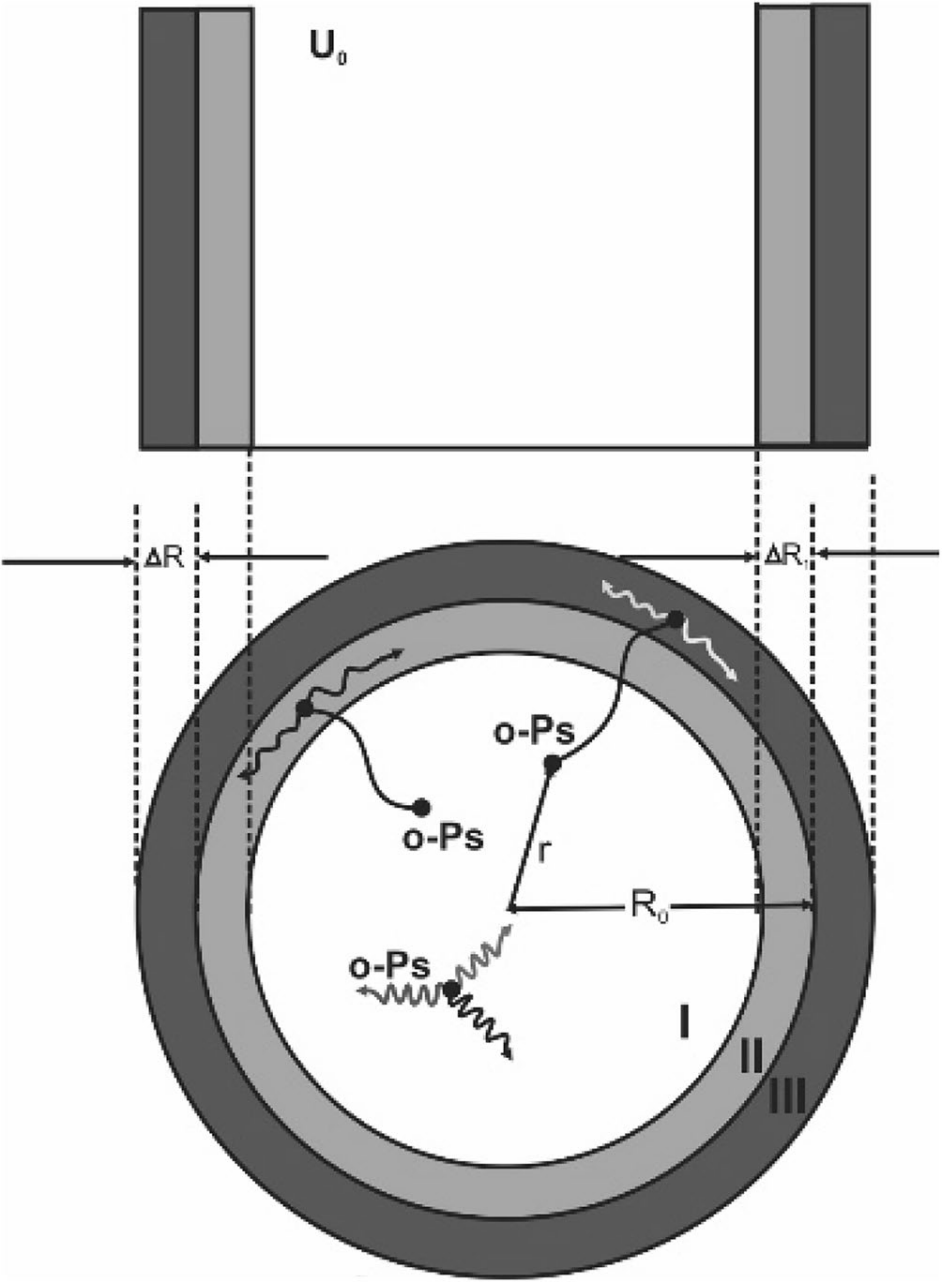
The annihilation of o-Ps in a pore (note that the electron density in region III is higher than that of region II).

o-Ps annihilation in a pore with any given shape, with pore volume V_0_ and surface are, S_0_, is modelled as a single unstable particle^12^ with mass of 2m_e_ (m_e_ is electron mass) confined in a finite spherical potential well, U(*r*), and radius R_0_. U(*r*) is zero for r between 0 and R_0_ − ΔR_1_ and is U_0_ for r between R_0_ − ΔR_1_ and R_0_ + ΔR. Here, r is distance from the central point of the sphere (pore), and R_0_ is effective pore radius defined as 3V_0_/S_0_. Due to the negative work function, o-Ps can be affected by the positive finite potential, U_0_ = − Φ_Ps_^21,46–48^, where Φ_Ps_ is the o-Ps work function. The potential well is given as:

1 $$\mathrm{U}\left(r\right)= \left\{\begin{array}{ll}0 & \quad if \;\; r<{\mathrm{R}}_{0}- \Delta {\mathrm{R}}_{1} \\ {\mathrm{ U}}_{0 } & \quad if \;\; {\mathrm{R}}_{0}- \Delta {\mathrm{R}}_{1}\le r\le {\mathrm{R}}_{0}+ \Delta R \end{array}\right.$$

where ΔR_1_ is an empirical value, which is assigned as 2.4a_0_^46^, the Bohr radius, a_0_ = 0.529 Å, and ΔR = 0.166 nm, is empirical value of the thickness of the electron layer surrounding a pore, which was introduced by the previous authors^13,19–21,48^ for both cases of the infinite and finite potential wells, this value is interpreted as an approximate value of the mean penetration depth of o-Ps in the bulk outside a pore. As mentioned above, o-Ps can undergo different annihilation characteristics in different regions of the pore, as illustrated in Fig. 1. When trapped in a pore with energy E ≤ − Φ_Ps_^47,48^, o-Ps is scattered multiple times by the pore surface becoming thermalized to undergo annihilation either via three gammas in the pore vacuum (3γ self-annihilation) of region I (*r* < R_0_ − ΔR_1_) with an annihilation rate^12,15^, λ_3γ_ = 0.00704 ns^−1^ or via pick-off annihilation in regions II (R_0_ − ΔR_1_ < *r* < R_0_) and III (R_0_ ≤ *r* < R_0_ + ΔR) with different rates. Those o-Ps states moved only in region I, they underwent self-annihilation (the relative contact density is considered as unit^15^). Those o-Ps states have not undergone self-annihilation in region I and enter regions II and III, can undergo pick-off annihilation. The pick-off annihilation rate in region II, which is caused by thermal vibration of atomics at the pore surface, is lower than that in region III, which is caused by a bulk electron. First, we consider those pores with radii exceeding ΔR_1_ and suppose that o-Ps is moving from the pore centre outwards (Fig. 1). Because of multiple scatterings, the initial o-Ps energy, E, therefore decreases until annihilation occurs. The average energy of o-Ps at the time of annihilation is denoted as E_av_(τ)^49^, where τ is the mean o-Ps lifetime (see S1). The radial o-Ps wavefunction, ${\psi }_{+}\left(r\right)$, can be obtained by deriving the Schrödinger equation with a finite spherical potential U(r). The wavefunction fractions of the o-Ps in regions II and III are used for calculation of the pore-related o-Ps lifetime that results in a surface-volume formula representing the dependence of the pore-related o-Ps annihilation rate on the surface-to-volume ratio, S_0_/V_0_, (or effective pore radius), and temperature as follows (for the derivation, see S1 and S2 of supplementary information):

2 $${\uplambda }_{\mathrm{o}-\mathrm{Ps}}={\lambda }_{3\gamma }+{\uplambda }_{0}(\mathrm{T})\frac{1}{2\mathrm{k}}\mathrm{\varkappa }\frac{{\mathrm{S}}_{0}}{{\mathrm{V}}_{0}}\left\{\upeta (\mathrm{T})[(1-\mathrm{exp}\left(-2\mathrm{k}{\mathrm{\Delta R}}_{1}\right)]+\mathrm{q}\frac{{\mathrm{S}}_{0}}{{\mathrm{V}}_{0}}\left[(1-\mathrm{exp}\left(-2\mathrm{k\Delta R}\right)\right]\mathrm{exp}(-2k{\mathrm{\Delta R}}_{1})\right\}$$

At room temperature, Eq. (2) is presented in terms of the effective pore radius as follows:

3 $${\uplambda }_{\mathrm{o}-\mathrm{Ps}}={\uplambda }_{3\gamma }+{\uplambda }_{0} \frac{1}{2\mathrm{k}}\mathrm{\varkappa }\frac{3}{{\mathrm{R}}_{0}}\left\{{\upeta }_{0}[(1-\mathrm{exp}\left(-2\mathrm{k}{\mathrm{\Delta R}}_{1}\right)]+\mathrm{q}\frac{3}{{R}_{0}}\left[(1-\mathrm{exp}\left(-2\mathrm{k\Delta R}\right)\right]\mathrm{exp}(-2\mathrm{k}{\mathrm{\Delta R}}_{1})\right\}$$

where ϰ and q are constants. ${\uplambda }_{0}(\mathrm{T})={\uprho }_{\mathrm{e}0}\left(\mathrm{T}\right)\uppi {\mathrm{r}}_{0}^{2}c$, where ${\mathrm{r}}_{0}$ is the classical electron radius (nm), T is temperature (K), *c* is the speed of light (nm/ns), λ_0_ is the value of λ_0_(T) at room temperature (T = 298 K), and ρ_e0_(T) is the average bulk electron density in region III at temperature T, η(T) is the ratio, ρ_eth_(T)/ρ_e0_(T), where ρ_eth_(T) is the average electron density arisen by the thermal atomic vibrations at the pore surface^21,33^ of region II at temperature T (η_0_ is the value of η(T) at room temperature, T = 298 K). Moreover, ${\text{k}} = \sqrt {4{\text{m}}\left( {{\text{U}}_{0} - {\text{E}}_{{{\text{av}}}} \left( \uptau \right)} \right)/\hbar^{2} }$. The quantity E_av_(τ), which was previously introduced by Nagashima et al.^49^, is an exponential decay function of time. We have shown in S1 that E_av_(τ) is also an exponential function of the minus ratio, − V/S. E_av_(τ) = U_0_exp(−μV/S) + E_th_, where μ is constant, and E_th_ ≈ (3/2)k_B_T_ps_, where T_Ps_ is o-Ps temperature (see S1). Note that ρ_e0_(T) linearly relates to the bulk density of the atoms, ρ_a0_(T), by a factor Z_eff_, an effective number of electrons^50^, namely, ρ_e0_(T) = Z_eff_ρ_a0_(T). As presented in S2, the values of four parameters, η_0_, ϰ, q, and μ, were semi-empirically found as follows: η_0_ ≈ 0.12, ϰ ≈ 0.057, q ≈ 0.03 nm, and μ ≈ 2.5 nm^−1^. Significantly, with given values of those parameters, the results of the SVF calculation are in good agreement with many other experimental data in the literature for pore sizes greater than ΔR_1_ (Tables 3, 4, 5). When the effective pore radius R_0_ becomes very large (in the range of the macropore), the results of the o-Ps annihilation rate calculated by SVF and by other models^12–20^ closely approach 0.00704 ns^-1^ because the fraction of the 3γ-intrinsic annihilation becomes dominantly and the fraction of the pick-off annihilation rate becomes very small. Therefore, for macropores, the difference in the o-Ps lifetimes related to the different pore sizes becomes difficult to significantly distinguish due to the overlaps in statistical deviations (Table 5). The SVF has been shown to be suitable for pores with different geometries and topologies. For those pores with effective radii smaller than ΔR_1_, the o-Ps annihilation rate is approximately λ_0_ (see S2). Note that the effective pore radius may differ from the geometrical radius. For example, for infinite cylindrical pores, the geometrical radius is two-thirds of the effective radius; however, for spherical pores, the geometrical radius is also the effective radius.

**Table 3:** Comparison of SVF, TE, and RTE calculations and PALS results for MCM-41, ZSM-5, and Al-Mil-53.

Sample	Geometry/topology	V/S (nm)	SVF (ns)	TE^13^ (ns)	RTE^19,20^ (ns), δ_RTE_ = 0.18 nm, *a* = 6 V/S (3D geometry)	Measured o-Ps lifetime (ns)
MCM-41	Channel	0.765	43.76	83.77	58.40 ***48.07***	43.40
ZSM-5	mfi topology	0.520	32.94 36.90^a^	44.93	38.20 ***24.25***	35.80
Al-Mil-53	Mil-53 topology	0.523	33.13	46.09	38.76 ***24.68***	32.20

^a^The result value calculated by using, λ_0_ = 1/τ_2_; data in bold italics indicate for 2D geometry of RTE calculation, *a* = 4 V/S.

**Table 4:** Comparison of the SVF, TE and RTE calculations and experimental results of micropores, MOF, from the literature^24^.

Sample	Geometry/topology	Side length of cubic pores, a_m_ (nm)	SVF (ns)	TE^13^ (ns)	RTE^19,20^ δ_RTE_ = 0.18 nm (ns)	Ref. of o-Ps lifetime (ns)
MOF-5^24^	*pcu*	1.008^24^	14.01	12.62	13.25	13.00^24^
IRMOF-20^24^	*pcu*	1.184^24^	17.38	18.60	18.43	20.00^24^
IRMOF-8^24^	*pcu*	1.101^24^	15.78	15.29	15.88	18.00^24^

**Table 5:** Comparison of SVF, TE and RTE calculations and large pores experimental results from the literature^14,57^.

Sample	Geometry/topology	Ref. of pore diameters, d_0_ (nm)	SVF (ns)	TE^13^ (ns)	RTE^19,20^ δ_RTE_ = 0.18 nm a = 2 (R + δ_RTE_) (ns)	Ref. of o-Ps lifetime (ns)
Silica gel^57^	–	4.00^57^	40.50	69.14	55.73	44.65^57^
Silica gel^57^	–	6.00^57^	55.60	108.04	74.43	60.20^57^
Silica gel^57^	–	10.00^57^	75.12	132.96	94.87	75.19^57^
Silica^57^	–	13.20^20^	86.18	138.47	104.06	84.25^20^
Zircon^14^	–	50.60^14^	121.15	141.97	131.19	122.70^14^
Silica^20^	–	136.14^20^	134.40	142.00	140.47	137.14^20^
Silica	–	280.49^20^	138.50	142.00	141.87	138.58^20^

## Discussion

### Pore structure-induced o-Ps annihilation explained by the SVF

Using the SVF can explain the influences of the bulk composition, structure, pore geometry and topology, as shown by the experimental results of this work and related literature. Generally, once positronium is formed, the positron lifetime spectrum must contain the following main components of lifetimes: the shortest component related to the positron annihilation in bulk averaged with the two-gamma p-Ps annihilation lifetime, the o-Ps annihilation in bulk material, the pore- and/or defect-related o-Ps annihilation lifetime, and the defect-related positron lifetimes. The last one, if it exists, may be merged with the o-Ps lifetime in bulk material. In this work, the positron lifetime spectra of all porous samples are deconvoluted into four lifetime components, τ_1_, τ_2_, τ_3_, and τ_4_, with the corresponding relative intensities, I_1_, I_2_, I_3_, and I_4_, as presented in Table 1, respectively. The first component, *τ*_1_, is ascribed to the average value of the positron lifetime in the bulk and the para-positronium lifetime (125 ps). The second component, τ_2_, relates to the bulk and ΔR-layer o-Ps lifetime, averaged with other components of bulk and (or) defect positron annihilations. In this study, the pore (and/or defect)-related o-Ps lifetimes are τ_3_ and τ_4_.

The bulk annihilation rate of o-Ps depends on the atomic density and effective electron number, Z_eff_^50,51^. The o-Ps annihilation rates in the bulk, λ_0_, can be^13,19,20^ 2 ns^−1^ or different from^52–54^ 2 ns^−1^. Experimental results show that the bulk composition also influences the o-Ps lifetime^22^. However, generally, the value λ_0_ ≈ 2 ns^−1^ is used for the SVF calculations (in fact, the measurements and deconvolutions using PALS show that the value 1/τ_2_ was observed to be approximately 2 ns^−1^). Therefore, in some cases, both values, λ_0_ = 2 ns^−1^ and ${\lambda }_{0}$ = 1/τ_2_, can be used for SVF calculation for reference and discussion.

The application of SVF is suitable to calculate the geometrical sizes of several cases of micropores with different geometries and topologies. Zeolites 5A and 13X each contain two micropores known as β-cages and α-cages^38^, which are truncated octahedrons and truncated cubic octahedrons^38^, respectively. The α-cages of zeolite 5A are connected through the window of the octagonal face, and those for zeolite 13 X are connected by the window of the dodecagonal face^38^. The o-Ps states trapped in those pores undergo annihilation with different o-Ps lifetimes, τ_3_ and τ_4,_ with intensities I_3_ and I_4,_ respectively. Noticeably, for the case of zeolite 5A, the side of the truncated octahedron is also the side of the truncated cubic octahedron, in which the side length of the truncated octahedron, 0.199 nm, calculated by SVF using τ_3_, approximates the values of the side length of the truncated cubic octahedron (0.198 nm), which is calculated using τ_4_. It is similar for the case of zeolite 13X. These results indicated that τ_3_ is related to the β-cage, and τ_4_ is associated with a larger α-cage. By application of the SVF, the values V/S calculated from τ_3_ and τ_4_ are convenient for calculations of pore radii and apertures of the 8-ring and 10-ring windows relevant to β-cages and α-cages. Namely, for the apertures of the 8-ring and 10-ring windows, the maximum diameters that molecules can diffuse along, estimated by using SVF, are 0.48 nm for zeolite 5A and 0.83 nm for zeolite 13X, which are reasonably in agreement with the results determined by other methods^38,55^. In contrast to zeolites 5A and 13X, a long lifetime (35.8 ns) was obtained for the ZSM-5 sample. The largest intensity value of I_4_ suggests that τ_4_ is associated with the predominant pore structure of ZSM-5, which must be the ZSM-5 framework. ZSM-5 is a hierarchical zeolite crystallized in an orthorhombic system with the *Pnma* space group^35,42^, and its framework contains sinusoidal channels intersected with straight channels structured by 10-member rings. The value of the pore diameter of the 10-member rings is within 0.52–0.57 nm, as estimated from τ_3_ (using SVF), which agrees with another report^35^. Therefore, the shorter lifetime component, τ_3_, is ascribed to the ZSM-5 microchannel. The long lifetime (35.8 ns) is attributed to the larger pores of the pore network of ZSM-5 with an effective pore radius of 1.57 nm. Similarly, for Al-Mil-53, the value of τ_3_ is ascribed to the micropore^56^ with an effective pore radius of 0.31 nm, equal effective radius of the orthorhombic pore with dimensions of 0.85 nm × 0.85 nm × 0.6 nm (diameter × diameter × height), and quite agreed with pore dimension data of the MIL-53*ht* (Al) micropore, as presented by Loiseau and Ferey et al.^34^. τ_4_ is associated with the effective pore radius, 1.59 nm, of the large pores of the Al-Mil-53 frame network. Note that the Mil-53 family can be synthesized in different crystal systems^34,56^. Using the SVF, one can calculate the o-Ps lifetimes associated with those cases of pores, and conversely, with given measured o-Ps lifetimes, one can calculate the pore size. For a pore with any shape, if the dimension of the pore is given, one can calculate the V/S ratio of that pore. Once the V/S ratio of a pore can be provided, the o-Ps lifetime associated with that pore can be calculated using SVF.

**Figure 2:**
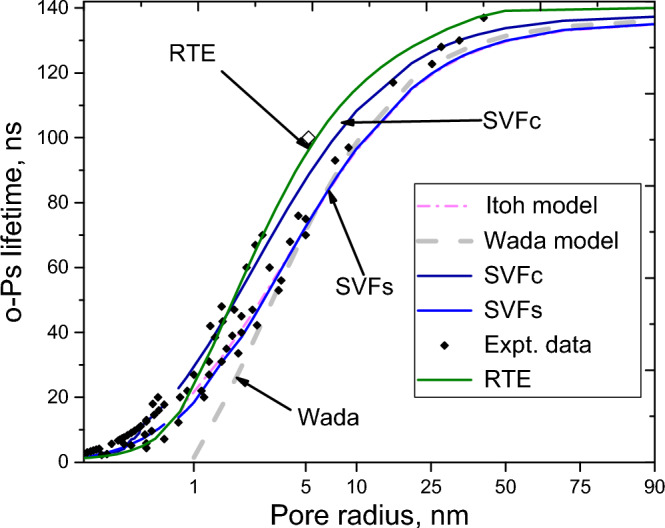
Comparison of the SVF and other models.

Furthermore, the SVF calculations are consistent with the experimental data obtained from both micro- and mesopores with different pore geometries and topologies, in which the SVF and TE and RTE calculations are approximately the same for the cases of micropores (Tables 3, 4, 5, and Fig. 2). For example, all SVF, TE and RTE models can appropriately predict the pore-related o-Ps lifetimes for micropores of metal–organic-frameworks with *pcu* topology, such as^24^ MOF-5, MOF-8, and MOF-20 (Table 4). However, the TE model fails for pores with radii larger than 1 nm. For many cases, as presented in Tables 3 and 5, the RTE calculations are not consistent with measured data for pores with diameters greater than 4 nm. The SVF calculations are in good agreement with the data of both micropores and mesopores (Tables 3, 4, 5). For macropores, the calculations of all models, SVF, TE, and RTE, closely approach 142 ns.

To visualize the comparison between the SVF and other models, we present in Fig. 2 the simulations of the RTE, Itoh, Wada, and the SVF models (SVF simulation includes SVFc and SVFs) plotted against the (geometrical) pore radius. Here, SVFc is the simulation of the SVF for infinite cylindrical pores, and SVFs is the simulation of the SVF for spherical pores, along with the experimental data, including the data from the literature^14,20,54^ and data of this work. For spherical pores, the geometrical radius is the same as the effective pore radius, and for infinite cylindrical pores, the geometrical radius (aperture) is approximately two-thirds of the effective pore radius. Because the pore shapes are not clarified in the set of the experimental data of the literature^14,20,54^, they may be the spherical, cylindrical, or other shapes. Therefore, we present the simulation for two typical pores: spherical and cylindrical shapes. Note that, based on the dependence of the o-Ps annihilation rate on the the S/V ratio, the SVF model can be used to simulate different pore shapes. In contrast, the Itoh and Wada models mainly consider spherical pores. Figure 2 indicates that SVFc (royal solid line) matches some experimental data, which are suggested to be associated with the cylindrical pores, while SVFs (blue solid line) matches some experimental data, which are suggested to be associated with spherical pores. The Itoh model is applicable to only pore radii larger than 0.8 nm. For pore radii larger than 0.8 nm, the simulation line of the Itoh model (red dashed dot line) almost coincides with the SVFs line. The Wada model is also applicable to only the cases of pore radii larger than 0.96 nm. For pores with radii larger than 2.5 nm, it is found that the Wada simulation (grey dashed line) almost merges with the SVFs and Itoh lines. For pore radii less than 2.5 nm, the Wada line significantly deflects from the experimental data. The RTE simulation (olive solid line), plotted against the values of modelling parameter, *a*/2 – δ_RTE_, which is interpreted by Gidley et al.^19^ and Dull et al.^20^ as pore radius.

Note that in Table 3, the RTE parameters, *a* (= *b* = *c*) = 6 V/S, are obtained from the relation, *l* = 4 V/S = (2/3)a^19,20^, the SVF and TE models use the effective pore radius equal 3 V/S for the calculations. In Table 4, the SVF and TE calculations also use the effective pore radius, R_0_, calculated from the infinite cubic rectangles (rectangular prism), while the RTE calculation use parameters, *a, b*, *c*, which are determined from the relation,* a* = *b* = *c* = 2(R_0_ + δ_RTE_)^19,20^, where δ_RTE_ = 0.18 nm, is the RTE constant (note that if one uses the value of the side length as the RTE parameter or uses values, *a* (= *b* = *c*) = 6 V/S, or *a* (= *b* = *c*) = 4 V/S the calculated o-Ps lifetimes using the RTE model for these cases are much less than the measured values). In Table 5, the SVF and TE models use the pore radius, d_0_/2 (d_0_ is the pore diameter), for the calculations, the RTE model applies the parameters, *a* = *b* = *c* = d_0_ + 2 δ_RTE_^19,20^.

It is shown in Fig. 2 that, in the range of micropores with radii smaller than 1 nm, the SFV, TE and RTE calculation results are approximately the same and are agreed with experimental results. For larger pores, the RTE simulation (in 3D geometry) significantly differs from the simulations of SVFs, Itoh, Wada, and many cases of the experimental data of literature, but keeps closely the SVFc simulation (cylindrical simulation) and some other experimental results. As seen in Tables 3 and 5, there are the agreements between some results of the RTE calculation results for micropores, such as, MOF-5, MOF-20, MOF-8, ZSM-5 and Al-Mil-53, with the experimental results. However, for many cases of mesopores as shown in Tables 3, 4, 5, there are discrepancies between the RTE calculation results and experimental results. This inconsistency can be explained by that the modelling parameter of RTE model, *a*, in the relation with the pore radius, *a* = 2(R + δ_RTE_), and that in the relation with the mean free path, *a* = (3/2)*l* = 6 V/S for 3D geometry^20^, or *a* = 4 V/S for 2D geometry^20^, appears not to be consistent for different cases of pore sizes and shapes (see the explanation in S3).

### Temperature dependence of the o-Ps annihilation lifetime

**Figure 3:**
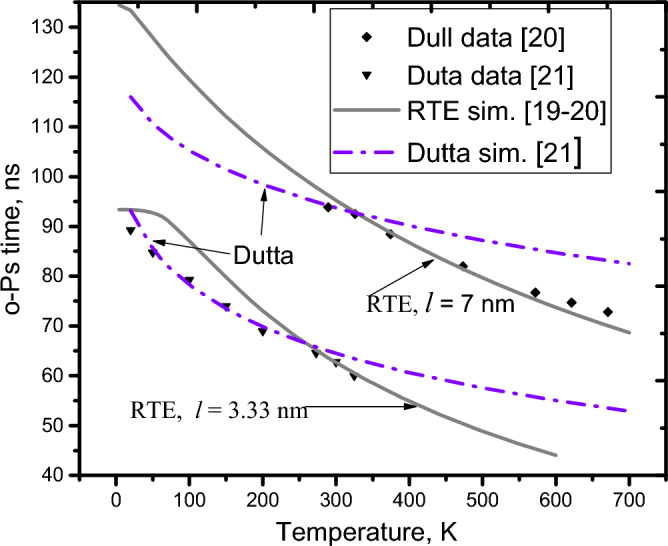
The characteristics of the o-Ps annihilation in the high temperature range (T > 273 K) appear to be different from those in the low temperature range (T < 273 K).

As reported**,** the o-Ps lifetime depends on the temperature^19–21^. However, this dependence has not been fully explained for a wide range of 273–700 K by any single formula^12–21^. For example, the RTE^19,20^ and Goworek^17^ models are only appropriate for high temperatures (T ≥ 273 K), while the Dutta et al. formula^21^ is suitable only for low temperatures (T ≤ 273 K), as shown in Fig. 3, in which the solid lines (*l* is the o-Ps mean free path, *l* = 4 V/S^19^) are simulated for the temperature dependence of the o-Ps lifetime using the RTE formula^19,20^, and the dashed-dotted lines are the simulation of the Dutta et al.^21^ formula. Using the SVF enables us to explain this fact (see more details in S3). First, considering the high temperature range (273–700 K), it is rational to assume that the averaged electron density in region II, ρ_eth_(T), linearly relates to the bulk electron density and to the mean square amplitude of the normal component of the thermal atomic vibrations^33^ at the pore surface, $\langle {\mathrm{u}}_{\mathrm{n}}^{2}(\mathrm{T})\rangle$:

4 $$\uprho_{{{\text{eth}}}} \left( {\text{T}} \right) = c_{1} \uprho_{{{\text{e0}}}} \left( {\text{T}} \right) + \left\langle {u_{n}^{2} \left( {\text{T}} \right)} \right\rangle$$

where *c*_1_ is the proportionality coefficient. As shown by previuos research^33^, in the range of high temperatures, the mean square amplitude is approximately proportional to k_B_T (T is the temperature in Kelvin, and k_B_ is Boltzmann’s constant). For convenience of presentation, $u1\equiv \langle {u}_{1n}^{2}(T)\rangle$ is denoted as the expression of the mean square amplitude of the normal component of thermal atomic vibrations at the pore surface for high temperatures (T ≥ 273 K). The following can be written:

5 $$\it u1 = c_{2} {\text{k}}_{{\text{B}}} {\text{T}} + \left\langle {u_{n0}^{2} } \right\rangle$$

where *c*_2_ is a proportionality factor and $\langle {\mathrm{u}}_{\mathrm{n}0}^{2}\rangle$ is defined as the mean square amplitude at low temperature T, for which k_B_T ≈ 0^33^. Thus:

6 $$\uprho_{{{\text{eth}}}} \left( {\text{T}} \right) = c_{1} {\text{p}}_{{{\text{e0}}}} \left( {\text{T}} \right)c_{2} {\text{k}}_{{\text{B}}} \left( {\text{T}} \right) + c_{1} \uprho_{{{\text{e0}}}} \left( {\text{T}} \right)\left\langle {u_{n0}^{2} } \right\rangle$$

**Figure 4:**
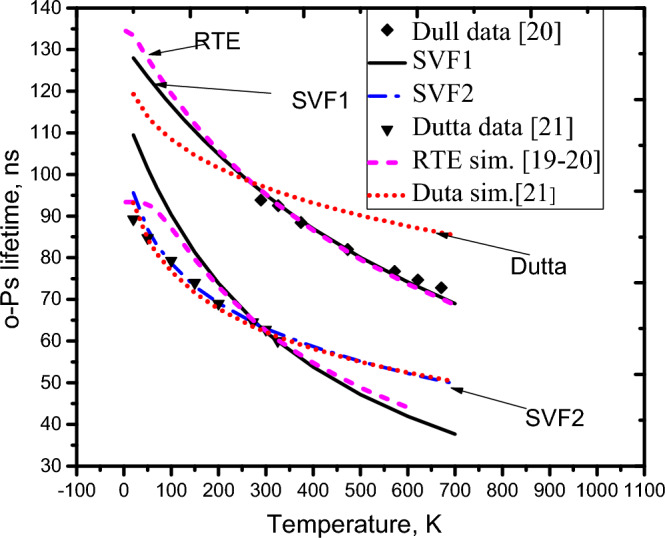
SVF1 simulation is consistent with the data of high-temperature (T ≥ 273 K) of the o-Ps lifetime, whereas SVF2 simulation is consistent with that of low-temperature (T ≤ 273 K).

η_1_(T) is denoted as the expression of η(T) for the high temperature range, T ≥ 273. Equation (6) becomes:

7 $$\upeta_{1} \left( {\text{T}} \right) = c_{1} c_{2} {\text{k}}_{{\text{B}}} {\text{T}} + c_{1} \left\langle {u_{n0}^{2} } \right\rangle$$

where c_1_ and c_2_ are proportionality coefficients. The values of $\langle {u}_{n0}^{2}\rangle$ and *u*_0_ = ${\left.u1\right|}_{\mathrm{T}=298\mathrm{ K}}$ are assigned as 0.00305 Å^2^ and 0.018 Å^2^, respectively (based on the experiences of the theoretical calculation^33^ and experiment^58^ for the case of SiO_2_). With given values of η_0_, *u*_0,_ and $\langle {u}_{n0}^{2}\rangle$, the values of *c*_*1*_ and *c*_*2*_ are therefore inferred (*c*_*1*_ ≈ 6.667 Å^−2^ and *c*_*2*_ = 0.582 Å^2^ eV^−1^). The expression of the SVF in using η_1_(T) is symbolized as SVF1. The SVF1 simulation (black solid line), as plotted in Fig. 4, shows agreement with the experimental data (♦ symbol) of the o-Ps lifetimes measured for high-temperature measurements^19,20^. The SVF1 simulation also agreed with the RTE simulation^19,20^ (short-dash lines) in this high temperature range. However, the SVF1 and RTE simulation results are not in line with the data obtained from the low-temperature measurements^21^ (20–273 K). This indicates that in the low temperature range, the expression of *u1* is no longer appropriate. To solve this problem, at low temperature (T ≤ 273), the mean square amplitude of the normal component of thermal atomic vibrations at the pore surface, *u2* ≡ $\langle {\mathrm{u}}_{\mathrm{n}2}^{2}(\mathrm{T})\rangle$, is  proposed to be approximately (see Fig. S2):

8 $$u2 = b\sqrt{{\mathrm{k}}_{\mathrm{B}}\mathrm{T}} + \langle {u}_{n0}^{2}\rangle$$

The expression of η(T) in the low temperature range, denoted as η_2_(T), can be written as:

9 $$\upeta_{2} \left( {\text{T}} \right) = c_{1} b\sqrt {{\text{k}}_{{\text{B}}} {\text{T}}} + c_{1} \left\langle {u_{n0}^{2} } \right\rangle$$

where b = 0.089 Å^2^(eV)^−1/2^, the proportionality coefficient that is estimated from the condition ${\left.u1\right|}_{\mathrm{T}=273\mathrm{ K}}={\left.u2\right|}_{\mathrm{T}=273 K}$). The simulation results of SVF2 (SVF2 is the expression of the SVF in using η_2_(T)), as plotted in Fig. 4 (blue dashed-dotted lines), agree with the low-temperature data of Dutta et al*.*^21^ (▼ symbol). The SVF2 is also consistent with the Dutta et al.^21^ simulation (short-dotted line). Generally, for the expression *un* = $\langle {\mathrm{u}}_{\mathrm{n}}^{2}\left(\mathrm{T}\right)\rangle$, the dependence of the mean square amplitude of the normal component of thermal atomic vibrations at the pore surface on a wide temperature range of 20–700 K can be well approximated by a polynomial of (k_B_T)^1/2^ variables, in which the 3rd-order approximation of u_n_(T) is as follows:

10 $${u}_{n} (T)={a}_{0}+{\mathrm{a}}_{1}{{(\mathrm{k}}_{\mathrm{B}}\mathrm{T})}^{1/2}+ {a}_{2}{\mathrm{k}}_{\mathrm{B}}\mathrm{T}+{a}_{3}{({\mathrm{k}}_{\mathrm{B}}\mathrm{T})}^{3/2}$$

**Figure 5:**
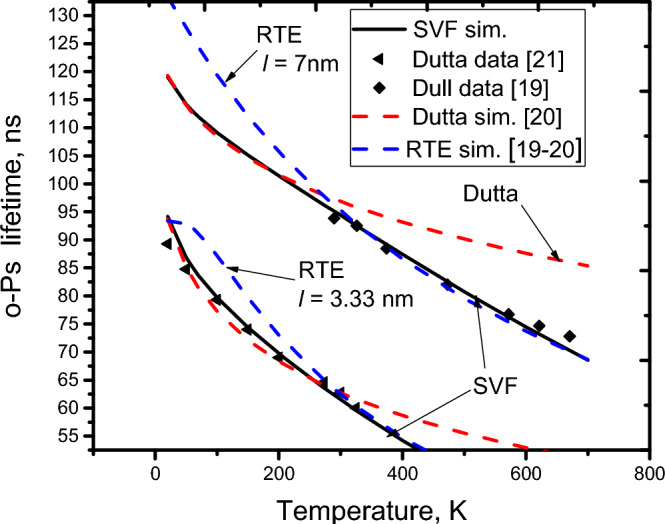
Comparisons of the o-Ps lifetimes calculated by SVF, RTE, and Dutta et al.^21^ with experimental data^19,21^.

**Figure 6:**
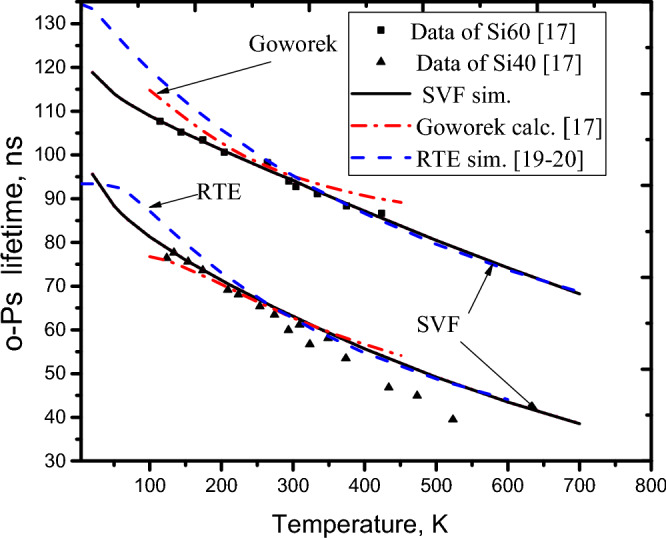
Comparison of the o-Ps lifetimes calculated by SVF and RTE, and Goworek et al*.*^17^ models with the experimental data^17^.

The values of the coefficients, *a*_*0*_ ≈ 0.00324 Å^2^, *a*_*1*_ ≈ 0.113 Å^2^(eV)^−1/2^, *a*_*2*_ ≈ − 0.561 Å^2^(eV)^−1^, and *a*_*3*_ ≈ 2.80 Å^2^(eV)^−3/2^, are easily given by fitting the right-hand side of Eq. (10) into *u1* and *u2* (see S3). The temperature simulations of the SVF using *un,* as plotted in Figs. 5 and Fig. 6, show agreement with both the high- and low-temperature data from the literature^17–21^. It is shown that the SVF can fully explain the dependence of o-Ps annihilation over a wide temperature range of 20–700 K, in which the calculation of the SVF (solid line) agrees with that of RTE^19,20^ (Dashed line) and Goworek et al.^17^ (Dash-dotted line) for temperatures greater than or equal to 273 K, while it is consistent with the Dutta et al.^21^ simulation for temperatures less than or equal to 273 K. It is noted that in the calculation of the o-Ps annihilation lifetimes using SVF presented above, the o-Ps temperature, T_Ps_, is approximately considered to be the sample temperature, T.

## Method

We performed both PALS and nitrogen adsorption measurements for MOF Al-Mil-53 (Nanochemazone), hierarchical zeolite ZSM-5 (ACS Materials, Si/Al = 38), and mesoporous MCM-41 (Sigma Aldrich). For the PALS measurement, powder samples with micron-sized particles were pressed into pellets at a pressure of approximately 1.5 GPa and heated for 10 h under vacuum at 100 °C. The PALS measurements were performed at room temperature (298 K) using an ORTEC fast–fast gamma coincidence spectrometer at the Center for Nuclear Techniques in Ho Chi Minh City with a lifetime resolution of 210 ps and with a Na-22 source of 30 μCi. The measurements were carried out under vacuum conditions at approximately 0.8 × 10^–4^ torr. Each obtained spectrum of positron lifetimes was accumulated to be 1 × 10^6^–4 × 10^6^ counts. PALS spectra of samples were deconvoluted using PALSfit^45^ into four components, τ_1_, τ_2_,* τ*_3_, and *τ*_4,_ with corresponding intensities, I_1_, I_2_, I_3_, and I_4_, respectively. The results are presented in Table 1. The specific surface area and pore volume of Al-Mil-53, ZSM-5 and MCM-41 were analysed using nitrogen isotherm adsorption^9,44^. The samples were heated for 5 h at 300 °C, and the adsorption measurements were conducted at 77 K using Micromeritics, Model 3Flex, version 3.02 (Hanoi National University of Education) and were analysed by using Brunauer-Emmett-Tel (BET)^9^ and t-plot analyses^44^; the results are given in Table 2.

## Conclusion

In summary, to investigate the o-Ps annihilation characteristics of nanoporous materials with different pore sizes, shapes, and topologies, in this work, we present both PALS and isotherm nitrogen adsorption measurements for different porous materials and an approach using a finite potential well for o-Ps annihilation in a pore. This results in the formula SVF that provides the calculation of the o-Ps annihilation rate that depends on the surface-to-volume ratio and temperature. The SVF calculation result is shown to agree with the experimental results of the positronium annihilation lifetimes obtained from the experiment of this work and from other data from the literature of both micro- and mesopores with different geometries and topologies. Using the SVF, we calculated the effective and geometrical sizes of microporous materials, 5A, 13X, ZSM-5, and Al-Mil-53, which are in good agreement with the results determined by other authors and methods. The experimental data of the temperature dependences of the pore-related o-Ps lifetime in a wide range of temperatures from 20 to 700 K are fully explained by the SVF. SVF can be applied for the pores formed in different shapes with the provision of the pore dimensions.

### Supplementary Information

Supplementary Information.

## Data Availability

The datasets used and/or analysed during the current study are available from the corresponding author on reasonable request**.**
