# Supplementary material for: Investigation of ortho-positronium annihilation for porous materials with different geometries and topologies
Source: Sci Rep. 2023 Aug 22;13:13707. doi: 10.1038/s41598-023-40901-3 (PMC10444843; doi:10.1038/s41598-023-40901-3)
Supplement: Supplementary file 1 — Supplementary Information. [file 41598_2023_40901_MOESM1_ESM.pdf]

Supplementary information

S1. The o-Ps energy

When trapped in a pore with an energy of a few electron-volts[1], o-Ps is scattered multiple times from atoms and molecules at the pore surface, thereby thermalizing and undergoing pick-off annihilation[2,3] with average energy, $E_{av}(\tau)$, τ is average lifetime of o-Ps. By the result of Nagashima et al.[3], the $E_{av}(\tau)$ has been found to monotonically decrease with o-Ps lifetime[3]. For o-Ps trapped in a pore, an increase of effective pore radius ($3V_0/S_0$, here V_0 and S_0 are pore volume and surface area, respectively) also results in an increase of the o-Ps lifetime. This means that $E_{av}(\tau)$ decreases as effective pore size increases. Therefore, $E_{av}(\tau)$ depends on V_0/S_0. In other words, $E_{av}(\tau)$ is a decreasing function of effective pore radius[3]. Normally, the value of $E_{av}(\tau)$ is small enough to expand $E_{av}(\tau)$ into 1st-order Maclaurin series of effective pore radius, R_0 ($R_0 = 3\,V_0/S_0$):

$$E_{av}(\tau) \approx E_{av}(\tau)|_{R_0\approx 0} + R_0 E'_{av}(\tau)|_{R_0\approx 0} \quad (1)$$

Differentiating Eq. (1) with respect to the variation of the effective pore radius R_0, results in:

$$d[E_{av}(\tau)] = \{E'_{av}(\tau)|_{R_0\approx 0}\}d(R_0) \quad (2)$$

Note that $E'_{av}(\tau)$ is found to be approximately proportional to minus $E_{av}(\tau)$[3]. This results in these following relations:

$$d\big(E_{av}(\tau)\big) = -\mu_1(T)E_{av}(\tau)d(R_0) \quad (2)$$

$$\frac{d\big(E_{av}(\tau)\big)}{E_{av}(\tau)} = -\mu_1(T)d(R_0) \quad (3)$$

where μ_1 is proportionality factor. Integrating differential equation, Eq. (3), gives:

$$E_{av}(\tau) = E_0\exp(-\mu_1(T)R_0) + E_{th} \quad (4)$$

where E_0 are constants in unit of eV. E_{th} is approximated the value of $E_{av}(\tau)$ with the very large value of R_0, $E_{th} = \lim_{R_0\to\infty} E_{av}(\tau)$. According to the result of Nagashima et al.[3], $E_{av}(\tau)$ approaches the value $(3/2)k_BT_{Ps}$ for increasing time, t, where k_B is Boltzmann constant, and T_{Ps} is o-Ps temperature. (o-Ps temperature, T_{Ps}, can differ from sample temperature[4], T). Therefore, E_{th} can be interpreted as, $E_{th} = (3/2)k_BT_{Ps}$. With very small value of effective pore radius, the value of $E_{av}(\tau)$ is approximated the value of the initial o-Ps energy, $E_{av}(\tau = 0) = -\Phi_{Ps}$[2,6] $= U_0$, Φ_{Ps} is o-Ps work function. Thus, $E_0 = U_0 - E_{Th}$ (particularly, for SiO_2, $U_0 = 1$ eV)[2]. Eq. (4) can be expressed by exponential function of V_0/S_0 as follows:

$$E_{av}(\tau) = E_0\exp(-\mu(T)V_0/S_0) + E_{th} \quad (5)$$

where $\mu(T) = 3\mu_1(T)$, in which μ is symbolled for the value of $\mu(T = 298\text{ K})$. The change of $E_{av}(\tau)$ with temperature is presented in S3.

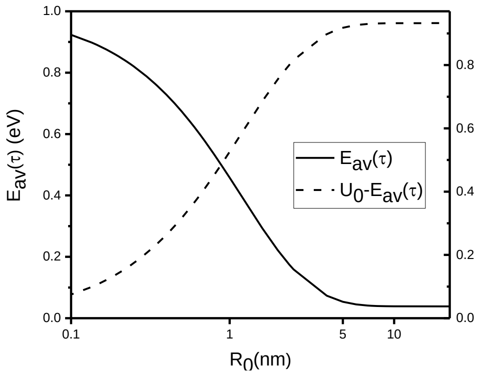

Fig. S1. The simulation of $E_{av}(\tau)$ varied with the effective pore radius.

S2. The derivation for wavefunction and annihilation rate of o-Ps

Supposed o-Ps being trapped in a pore, the o-Ps lifetime monotonically increases with pore volume[5,10], and decreases with pore surface area[5,8]. However, even with the same value of pore volume, a pore can have different surface areas in different pore shapes (e.g., cubic, and spherical pores). As a result, the lifetimes of the o-Ps states in different pores with the same volume and with different surface areas, are different. Considering the o-Ps states in different pores with different values of pore volumes and surface area, we assume that the lifetimes of o-Ps states in those pores with the same value of V_0/S_0, have the same value (without taking into account the role of pore surface composition). Based on this assumption, we presented the model for describing o-Ps annihilation in a pore as shown by Eq. (1) and Fig. 1 of main text. In the spherical polar coordinate, the radial function, $\psi_+(r)$, of the o-Ps spherical wavefunction can be obtained from deriving the Schrödinger equation with a finite potential, U(r) as presented in main text. The o-Ps radial wavefunctions are different for different regions of a pore. In region I ($r < R_0 - \Delta R_1$), the radial wavefunction of the o-Ps is approximated by the ground state wave function of o-Ps as follows (see ref. 7 for example):

$$\psi_{1+}(r) = \alpha\frac{\sin(k_0 r)}{r} \quad (6)$$

where α is a factor independent on r, $k_0 = \sqrt{4m_eE_{av}(\tau)/\hbar^2}$, m_e is the electron mass, and $\hbar$ is the reduced Planck's constant. For $R_0 - \Delta R_1 \leq r \leq R_0$, the radial wavefunction of the o-Ps is approximated as follows:

$$\psi_{2+}(r) = \beta_0 \frac{\exp\{-kr_1\}}{r_1} \tag{7}$$

where $r_1 = r - (R_0 - \Delta R_1)$, β_0 is a factor independent on r, and, $k = \sqrt{4m_e(U_0 - E_{av}(\tau))/\hbar^2}$. The o-Ps can undergo pick-off annihilations in both regions II and III. For those o-Ps that have not annihilated in region II or moved back to region I for the self-annihilation, they penetrate the region III ($R_0 \le r \le R_0 + \Delta R$) with a nonzero probability. The radial wavefunction of o-Ps in region III, $\psi_{3+}(r)$, is therefore satisfied this following relation:

$$|\psi_{3+}(r)|^2 = D^2|\psi_{2+}(r)|^2 \tag{8}$$

where coefficient, D^2, is the fraction of o-Ps entering region III from region II. It is noted that the coefficient, D^2, arises only for the case of unstable o-Ps (for the case of stable particle such as electron, the wavefunction in region III is the same with that as in region II). We will consider below the coefficient, D^2, which depends on the pore surface area and volume. Here, we deal with o-Ps, which have no angular momentum, the azimuthal and magnetic quantum numbers, l_0 and m_0 are all zero, the spherical harmonic[7], $Y_{lm}(\theta, \varphi) = (1/4\pi)^{1/2}$). Neglecting the o-Ps escaping from pores, the pick-off annihilation rate, $\lambda_{pick-off}$, can be expressed as follows[5,8]:

$$\lambda_{pick-off} = \pi r_0^2 c\beta_0^2 \int_0^{\Delta R_1} \rho_{eth}|\psi_{2+}(r)|^2\, r_1^2 dr_1 + \pi r_0^2 c\beta_0^2 D^2 \rho_{e0}(T) \int_{\Delta R_1}^{\Delta R_1+\Delta R} |\psi_{2+}(r)|^2\, r_1^2 dr_1 \tag{9}$$

where r_0 is the classical electron radius (nm), and c is the speed of light (nm/ns), $\rho_{e0}(T)$ is the average bulk electron density (nm^{-3}) in the region III (bulk density of electron), and $\rho_{eth}(T)$ is the average electron density (nm^{-3}) in region II arisen by the thermal atomic vibrations at the pore surface[9,10] at temperature T. Note that $\rho_{e0}(T)$ linearly relates to bulk density of the atoms, $\rho_{a0}(T)$, by a factor Z_{eff}, an effective number of electrons[11], $\rho_{e0}(T) = Z_{eff}\rho_{a0}(T)$. Eq. (9) can be re-written as:

$$\lambda_{pick-off} = \lambda_0(T)\beta_0^2 \int_0^{\Delta R_1} \eta(T)|\psi_{2+}(r)|^2\, r_1^2 dr_1 + \beta_0^2 D^2 \lambda_0(T) 4\pi \int_{\Delta R_1}^{R_0+\Delta R} |\psi_{2+}(r)|^2\, r_1^2 dr_1 \tag{10}$$

where $\lambda_0(T) = \rho_{e0}(T)\pi r_0^2 c$ (ns^{-1})[8,10,11], being the bulk annihilation rate of o-Ps at temperature, T(K); $\eta(T)$ is the ratio of $\rho_{eth}(T)/\rho_{e0}(T)$. The value of k depends on the value of $E_{av}(\tau)$, which in turn, depends on the effective pore radius and temperature. The expression of, $\lambda_{pick-off}$, which is obtained by integrating right hand-side of Eq. (10), is as follows:

$$\lambda_{pick-off} = \lambda_0(T)\, \frac{1}{2k}\beta_0^2\{\eta(T)[(1 - \exp(-2k\Delta R_1)] + D^2[(1 - \exp(-2k\Delta R)]\exp(-2k\Delta R_1)\} \tag{11}$$

At room temperature, the Eq. (11) becomes:

$$\lambda_{pick-off} = \lambda_0\, \frac{1}{2k}\beta_0^2\{\eta_0[(1 - \exp(-2k\Delta R_1)] + D^2[(1 - \exp(-2k\Delta R)]\exp(-2k\Delta R_1)\} \tag{12}$$

where η_0 is the value of $\eta(T)$ at the room temperature, T = 298 K. The pore related annihilation rate of o-Ps with taking into account the 3γ self-annihilation rate, $\lambda_{3\gamma}$, is expressed as[12]:

$$\lambda_{o-Ps} = \kappa\lambda_{3\gamma} + \lambda_{pick-off} \tag{13}$$

where κ explained as relative contact density[12] is applied for the correction of the 3γ self-annihilation rate, $\lambda_{3\gamma}$, of o-Ps in the medium. For small void, the value of $\kappa\lambda_{3\gamma}$ is negligible, while for large pore, κ is approximately unit[12].

Consider to β_0^2, provided that the unit of o-Ps annihilation rate in Eq. (11) is ns^{-1}, the dimension of β_0^2 should be nm^{-1}. Furthermore, for $R_0 > \Delta R_1$, substituting $r = R_0 - \Delta R_1$ into Eq. (7) gives

$$P(R_1) = |\psi_{2+}(R_0 - \Delta R_1)|^2 = \frac{\beta_0^2}{(R_0 - \Delta R_1)^2} \tag{14}$$

where $P(R_1)$ is an expression relevant to the probability of finding o-Ps at position, $R_1 = R_0 - \Delta R_1$. Provided that $P(R_1)$ has defined, nonzero value, β_0^2 must be in this following expression:

$$\beta_0^2 = f_1(R_1)(R_0 - \Delta R_1)^2 \tag{15}$$

and, therefore:

$$P(R_1) = f_1(R_1) \tag{16}$$

where $f_1(R_1)$ is expression of R_1 having defined and nonzero value when $R_1 = 0$. Note that the greater the pore effective radius, R_1, the higher the 3γ self-annihilation rate, the lower the pick-off annihilation rate of o-Ps, therefore, the lower the probability of finding o-Ps at R_1, therefore, $f_1(R_1)$ inversely varies with V_1, the volume of sphere with radius R_1. Referring to the dimension of β_0^2, the expression, $f_1(R_1)$, can be set as below:

$$f_1(R_1) = \varkappa_1 f_2(R_1)/\, V_1 \tag{17}$$

such that $\varkappa_1$ is constant, and the dimensionless factor, $f_2(R_1) = 1$, when R_1 is very large. Eq. (15) becomes:

$$\beta_0^2 = \frac{3}{4\pi}\varkappa_1 f_2(R_1)/\, R_1 \tag{18}$$

Provided that $f_1(R_1)$ has the defined and nonzero value when $R_1 = 0$, the value of $f_2(R_1)$ must vanish when $R_1 = 0$. It is allowed to assume that $f_2(R_1)/R_1 \approx 1/R_0$. Therefore:

$$\beta_0^2 = \frac{3\varkappa_1}{4\pi R_0} \approx \varkappa S_0/V_0 = 3\varkappa/R_0 \tag{19}$$

where $\varkappa = \varkappa_1/4\pi$, being constant, S_0 and V_0 are respectively the surface area and volume of a pore with effective radius, R_0.

Considering the coefficient D^2, it is noted that an increase in volume results in a decrease in D^2; and with a given volume, V_0, an increase in the pore surface area, S_0, results in an increase in the pick-off annihilation rate in region III. Therefore, D^2 is decreasing function of S/V. In the first order approximation, D^2 is expressed as

$$D^2 \approx qS_0/V_0 + D_0 \tag{20}$$

where q is a coefficient in nm, $D_0 = D^2|_{\frac{S}{V}=0} = 0$. Let to assume that the relative contact density, $\kappa = 1$, for $R_0 > \Delta R_1$, the pore related o-Ps annihilation rate, λ_{o-Ps}, is calculated using Eq. (13) as follows:

$$\lambda_{o-Ps} = \lambda_{3\gamma} + \lambda_0(T)\,\frac{1}{2k}\varkappa\frac{S_0}{V_0}\Big\{\eta(T)[(1-\exp(-2k\Delta R_1)] + q\frac{S_0}{V_0}[(1-\exp(-2k\Delta R)]\exp(-2k\Delta R_1)\Big\} \tag{21}$$

or:

$$\lambda_{o-Ps} = \lambda_{3\gamma} + \lambda_0(T)\,\frac{1}{2k}\varkappa\frac{3}{R_0}\Big\{\eta(T)[(1-\exp(-2k\Delta R_1)] + q\frac{3}{R_0})[(1-\exp(-2k\Delta R)]\exp(-2k\Delta R_1)\Big\} \tag{22}$$

At room temperature, the Eq. (20) becomes:

$$\lambda_{o-Ps} \approx \lambda_{3\gamma} + \lambda_0\,\frac{1}{2k}\varkappa\frac{S_0}{V_0}\Big\{\eta_0[(1-\exp(-2k\Delta R_1)] + q\frac{S_0}{V_0}[(1-\exp(-2k\Delta R)]\exp(-2k\Delta R_1)\Big\} \tag{22}$$

and Eq. (21) becomes:

$$\lambda_{o-Ps} \approx \lambda_{3\gamma} + \lambda_0\frac{1}{2k}\varkappa\frac{3}{R_0}\Big\{\eta_0[(1-\exp(-2k\Delta R_1)] + q\frac{3}{R_0}[(1-\exp(-2k\Delta R)]\exp(-2k\Delta R_1)\Big\} \tag{23}$$

where λ_0 is the value of $\lambda_0(T)$ at room temperature, T = 298 K. Eq. (18) is meaningful and applicable only for those cases with pore radii greater than ΔR_1. For $R_0 = \Delta R_1$, the fraction of 3γ self-annihilation of the o-Ps can be negligible[12], almost o-Ps undergo pick-off annihilation with electron of the pore surface. Due to relative contact density, κ, is negligible[12] for these cases, the following condition is satisfied:

$$\Big\{\int_0^{\Delta R_1}[\psi_{2+}(r)]^2\, r_1{}^2 dr_1 + \int_{\Delta R_1}^{\Delta R_1+\Delta R} D^2|\psi_{2+}(r)|^2 r_1{}^2 dr_1\Big\} \approx 1 \tag{24}$$

The condition of Eq. (24) provided that at room temperature, and for the case of a pore with effective pore radius equal ΔR_1, the o-Ps annihilation rate is λ_0, which in fact, is approximately 2 ns^{-1} for many materials. For those pores with pore radii smaller than ΔR_1, all equations, Eq. (6) - Eq. (12), and Eq. (14) – Eq. (23) are unapplicable. However, this following condition is held:

$$\int_{R_1}^{R_2}[\psi_+(r)]^2\, r^2 dr \approx 1 \tag{25}$$

where $R_2 = R_0 + \Delta R_1$, $\psi_+(r)$ is the radial part of a normalized wavefunction of the o-Ps in a pore with effective radius smaller than ΔR_1. The expression of $\psi_+(r)$ may differ from that of the case with pore radius greater than ΔR_1 that presented above. In practice, the value of ΔR_1 is very small (ΔR_1 is about $2.4a_0$)[13], and the bound of e^+-e^- cannot exist in the dispace smaller than[8] 0.097 nm. It is supposed that o-Ps formed and existed in the region, 0.097 nm – $2.4a_0$, behaves as if it does in the bulk materials, and o-Ps annihilation rates are approximate λ_0. It is noteworthy to found that the RTE model also gives similar result. The RTE model can be applied only for those values of the pore-size-related parameter, "*a*", defined by Gidley et al.[5] and Dull et all.[14], greater than or equal 0.36 nm. Namely, when, "*a*" = 0. 36 nm, the pore diameter is zero, and RTE calculation gives the result of o-Ps annihilation rate approximates 2 ns^{-1}. The similar situation occurs with the TE model[15]. The TE calculation gives the annihilation rate be to 2 ns^{-1} when pore size approaches zero. These clearly show that at room temperature, the bulk o-Ps annihilation rate is 2 ns^{-1} interpreted by the TE and RTE models, and that is λ_0, in the interpretation of SVF.

Using condition of Eq. (24) and applying the experimental results of o-Ps lifetimes associated with porous samples, MCM-41, ZSM-5, and Al-Mil-53 as shown in Table 1, along with the values of pore volumes, surface areas associated with these porous sample as shown in Table 2 to Eq. (22) and Eq. (23) results in four transcendental equations of four parameters, η_0, $\varkappa$, q, and μ, which turn out to be consistent. The calibration of SVF into experimental results accomplished by deriving those equations gives, $\eta_0 \approx 0.12$, $\varkappa = \varkappa_1/4\pi = 0.716/4\pi = 0.057$, $q \approx 0.03$ nm, $\mu \approx 2.5$ nm^{-1}. Notably, the value of, $\eta_0 = 0.12$, agrees with the theoretical calculation[13,16],

It is noted that for the calculus using radial variable, r, the role of the parameters, k, $E_{av}(\tau)$, and V/S can be clarified as follows. For the SVF model as described by Eq. (1) and Fig. 1 of main text, the pore with volume, V_0, and with surface area, S_0, and with any shape, is modelled as a spherical pore with pore radius, $R_0 = 3V_0/S_0$ (the dimension is in nm). The value of R_0 is defined as an effective pore radius. The value, $3V_0/S_0$, is measured by gas adsorption method. In spherical coordinate, the radian variable, r, the distance of o-Ps from pore center, defines the radial position of o-Ps. The dimension of r (nm) is the same dimension of R_0 $=3V_0/S_0$ (nm). The value of r is varied from 0 to ($R_0 + \Delta R$). However, r is a variable specifying the radial position of the moving o-Ps, while $3V_0/S_0$ is not variable in the calculus of the integration or differentiation, it is a constant value for a given pore size. The value of $E_{av}(\tau)$ and k are functions of V_0/S_0 of different pore sizes, but they are constants for a given pore size (it is not function of r). The variable, r, is used to calculate the integration or differentiation, etc. to obtain the analytical expressions of o-Ps annihilation rate for a given pore size. While taking the integral or differentiate, r is considered as variable, but R_0, k, $E_{av}(\tau)$, and V_0/S_0 (of a given pore) are considered as constant. Therefore, in the formula of SVF, there are only values of R_0 and V_0/S_0 related to the o-Ps annihilation rate.

S3. Appendix

The explanation for the RTE calculation results presented in Tables 3, 4, 5. The RTE model[5] use modeling parameters, *a, b, c,* for calculations of the o-Ps lifetimes. Therefore, in the RTE model, the o-Ps lifetime depends on these modeling parameters, *a, b, c*, but not directly depends on the pore size. Gidley et al.[5] and Dull et al.[14] have provided the relation of these parameters, *a (or b, or c),* and pore radius, $a = 2\ (R + \delta_{RTE})$, where R is the pore radius (nm), δ_{RTE} = 0.18 nm, being the RTE constant, and the connection of these parameter, *a (*and *b,* and *c)*, with mean free path, $a = l/2$ for 1D geometry, $a = l = 4$ V/S for 2 D geometry, and $a = (3/2)l = 6$ V/S for 3D cubic geometry. Hence, it is possible to specify different solutions to define the RTE parameters, *a, b, c*.

In Table 3, the values of these parameters (3D cubic geometry, $a = b = c$, are determined from the value of mean free path, l = 4 V/S, and, $l = (2/3)a$. For the RTE calculation in these cases, it is inconsistently for defining the geometry to calculate the RTE parameter, *a*. For example, for the case of MCM-41, the chosen geometry of 2D seems to be better than that of 3D. However, for the cases of ZSM-5 and Al-Mil-53, the chosen geometry of 3D seems to be better than that of 2D. Note that in Table 3 of main text, in addition to 3D calculation results, we indicate the values of RTE calculation for 2D geometry in *bold italic data*, for reference.

In Table 4, if one uses the value of side length of cubic pore, a_m, as RTE parameter, *a*, the RTE calculation results are much less than the experimental results; and, it is seemly that the relation, $a = 4$ V/S for the case of 2D geometry, or $a = 6$ V/S for the case of 3D geometry, are both not suitable solution for the RTE calculation for the cases of MOF-5, MOF-20, and MOF-8. Below is the explanation for this inadequacy.

Consider to the calculation of RTE model for the cases of MOF-5, MOF-20 and MOF-8 (Table 4). According to the definitions of the RTE modeling parameters[5,14], *a, b, c*, one can specify the different relations between the RTE parameter, *a*, and the pore size parameter for a given pore, and one can calculate the different values of these parameters, $a\ (= b = c)$, and therefore, the different results of o-Ps lifetime can be obtained from RTE calculations for a given pore. This inconsistency is illustrated by different case below:

i) Case 1. Assigning the value of the side length, a_m, to modeling parameters, *a*, $a_m = a\ (= b = c)$, the results of o-Ps lifetimes calculated by RTE model presented in Table S3.1a, which are much less than experimental results. Hence, this solution is not suitable for calculation of parameter, *a* from the value of cubic side, a_m.

ii) Case 2. Using the equality[5,14], $a\ (= b = c) = 6$ V/S, where, V/S ≈ $a_m/4$, is the volume-to-surface area ratio of infinite rectangular prism, with sides, $a_m = b_m$ = side length of pore, and side, c_m, is infinite. For this case, the results of o-Ps lifetimes calculated by RTE model are presented in Table S3.1b, which are deferent with experimental results. This solution is not good one for calculation of parameter, *a*, from the value of cubic side, a_m.

iii) Case 3. Using, $a\ (= b = c) = 2(R + \delta_{RTE})$, where R is pore radius of circumscribed sphere of cubic pore with a side length, a_m, the o-Ps lifetimes calculated by RTE model are presented in Table S3.1c, relatively differ from experimental results. This solution is not best one for the calculation of parameter, *a*.

iv) Case 4. Using, $a\ (= b = c) = 2\ (R_0 + \delta_{RTE})$, where R_0 is effective pore radius, R_0 = 3V/S as described in the case ii), the results of o-Ps lifetimes are presented in Table S3.1d, which are consistent with experimental results.

Table S3.1a. Comparison of the RTE calculation results and the experimental results of Crivelli et al.[18], in which the RTE modeling parameter, $a = a_m$.

Sample	Side length of pore (nm) $a_m = b_m = c_m$	RTE parameters ($a = b = c$ = side length) (nm)	RTE[5,14] δ_{RTE} = 0.18 nm (ns)	Ref. of o-Ps lifetime (ns)
MOF-5[18]	1.008[18]	1.008	2.50	13.00[18]
IRMOF-20[18]	1.184[18]	1.184	3.84	20.00[18]
IRMOF-8[18]	1.101[18]	1.101	3.16	18.00[18]

Table S3.1b. Comparison of the RTE calculation results and the experimental results of Crivelli et al.[18], in which the RTE modeling parameter, $a = 6$ V/S, where V/S is explained as in the text of case ii).

Sample	Side length of pore (nm) $a_m = b_m$, c_m is infinite	Calculated V/S (nm)	RTE parameters (a = b = c = 6 V/S) (nm)	RTE[5,14] δ_{RTE} = 0.18 nm (ns)	Ref. of o-Ps lifetime (ns)
MOF-5[18]	1.008[18]	0.252	1.512	7.48	13.0[18]
IRMOF-20[18]	1.184[18]	0.296	1.776	11.54	20.0[18]
IRMOF-8[18]	1.101[18]	0.275	1.652	9.51	18.00[18]

Table S3.1c. Comparison of the RTE calculation results and the experimental results of Crivelli *et al.*[18], in which the RTE modeling parameter, *a*, determined from (geometrical) diameter of the circumscribed sphere of cubic pore.

Sample	Side length of pore (nm) $a_m = b_m = c_m$	Diameter of circumscribed sphere, d_0, (nm)	RTE parameters ($a = d_0 + 0.36$ nm) (nm)	RTE[5,14] δ_{RTE} = 0.18 nm (ns)	Ref. of o-Ps lifetime (ns)
MOF-5[18]	1.008[18]	1.79	2.11	17.89	13.00[18]
IRMOF-20[18]	1.184[18]	2.05	2.41	24.14	20.00[18]
IRMOF-8[18]	1.101[18]	1.92	2.27	21.21	18.00[18]

Table S3.1d. Comparison of the RTE calculation results and the experimental results of Crivelli *et al.*[18], in which the RTE modeling parameter, *a*, determined from effective pore radius of the circumscribed sphere of cubic pore.

Sample	Side length of pore (nm) $a_m = b_m$ c_m is infinite	Effective pore radius, $R_0 = 3V/S$ (nm)	RTE parameters $a = b = c = 2\ (R_0 + 0.18\ nm)$ (nm)	RTE[18,19] $\delta_{RTE} = 0.18$ nm (ns)	Ref. of o-Ps lifetime (ns)
MOF-5[18]	1.008[18]	0.756	1.872	13.25	13.0[18]
IRMOF-20[18]	1.184[18]	0.888	2.136	18.43	20.0[18]
IRMOF-8[18]	1.101[18]	0.825	2.010	15.88	18.00[18]

The o-Ps lifetime calculated by the RTE model using the case 4, which is presented in Table S3.1d, are best results by means of the best agreement with the experimental results. It is noted that the assumption that o-Ps can move along the infinite rectangular prism, which result in in the calculations of all SVF, TE, and RTE models consistent with experimental results for micropores of MOF, agrees with discussion of Dutta et al.[17]. Nevertheless, although, the solution iv) is the best one for the RTE calculations of MOF-5, MOF-20, and MOF-8, it is not good solution for the cases of pores presented in Table 5. In Table 5, the RTE parameter, *a* (= *b* = *c*) = 2(R + δ_{RTE}), where R is pore radius referred from literature[19,20]. Using the values of *a* calculated from that relation, the RTE calculation results are not agreed with experimental results of literature[14,19,20].

The mean square amplitude of normal component of the thermal atomic vibrations. Firstly, we consider temperature dependence of o-Ps lifetime for high temperature range. In this region, we can determine the expression of *u1*, the mean square amplitude of normal component of the thermal atomic vibrations that approximately depends on the k_BT. We apply this result to calculate the o-Ps lifetimes using SVF for different temperatures and compare these results with experimental results published by other authors[10,17]. It is observed that these results of the calculated o-Ps lifetimes agree with experimental results of o-Ps lifetimes measured at vacuum condition and for different high temperatures. Noticeably, for high temperature, the changes of o-Ps lifetimes calculated by SVF, are agreed with those of the o-Ps lifetimes calculated by RTE. However, for low temperature, the application of *u1* to calculate the changes of o-Ps lifetimes using SVF are not consistent with experimental results reported by Dutta et al.[10] The o-Ps lifetimes calculated by RTE for low temperature also are inconsistent with data of Dutta et al.[10] Referring to the result of Dutta et al.[10], we propose another expression, *u2*, the mean square amplitude of normal component of the atomic vibrations, that approximately depends on the $(k_BT)^{1/2}$ for the low temperatures. The application of *u2* to calculate the o-Ps lifetimes using SVF for different low temperature results in consistency of the SVF calculated o-Ps lifetimes and experimental results of Duta et al.[10] Several values of *u1* and *u2* are calculated and plotted against $(k_BT)^{1/2}$ in Fig. S2.

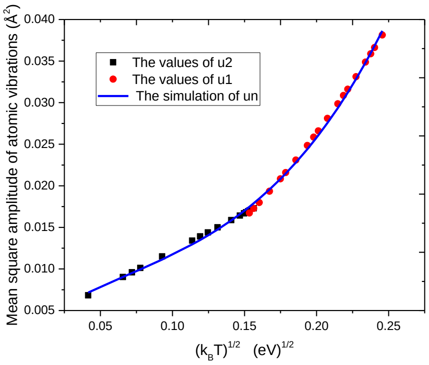

Fig. S2. Several values of *u1* and *u2* are calculated and plotted against $(k_BT)^{1/2}$; the blue solid line is simulation of *un* that differs from *u1* and *u2* with mean relative standard deviation of 0.017.

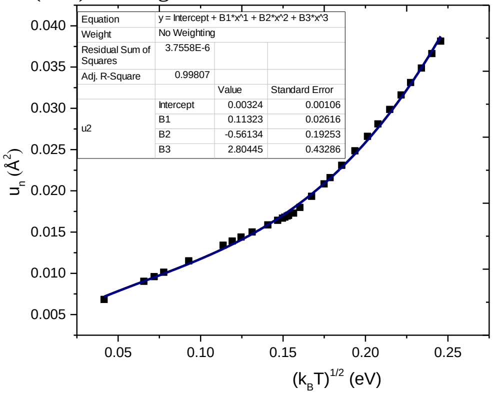

Fig. S3. The mean square amplitude of normal component of the thermal atomic vibrations, *un*, can be approximated by 3rd polynomial of $(k_BT)^{1/2}$ variables

It is noted that the presentation of *u1* and *u2* is only the intermediate stage, our purpose is to search for the general expression of *un*, the mean square amplitude of normal component of the thermal atomic vibrations adequate for at all temperature. In general, its analytical expression is not well known, however, it manifests itself that *un* approximately depends on k_BT for the high limit of temperatures, and $(k_BT)^{1/2}$ for the low limit of temperature. This suggests us to approximate it by polynomial of $(k_BT)^{1/2}$ variables. The values of parameters related to 3rd polynomial described in Eq. (10) of main text, a_0 = 0.00324 Å^2, a_1 = 0.113 Å^2(eV)$^{-1/2}$, a_2 = - 0.561 Å^2(eV)$^{-1}$, and a_3 = 2.80 Å^2(eV)$^{-3/2}$, are numerically calculated by fitting *un* into the calculated values of *u1* and u2 (Fig. S3. shows the results of this fitting that is simply and reliably to return the fitting coefficients). In Fig. S2, the simulation of *un* (blue solid line) shows to be well fitted with the values of *u1* and *u2*: the simulation of *un* differs from *u1* and *u2* with mean relative deviation of 0.017.

Noticeably, in Fig. 3-6, the RTE simulation uses the RTE parameter, *a* = (3/2)*l*, where *l* = 7 nm and *l* = 3.33 nm; while, in Fig. 3-5, Dutta simulations[21] are applied for pore radii, R_d = 7. 2 nm and R_d = 2.76 nm. In Fig. 4, SVF1 is simulated by SVF for cylindrical shape with pore aperture, R_c = 5.95 nm, and for high temperatures, T ≥ 273 K, SVF2 is done so for R_c = 2.55 nm, and for low temperature, T ≤ 273 K. In Fig. 5, SVF simulates for cylindrical pore with pore apertures, R_c = 5.95 nm, and 2.55 nm, and for temperature range of 20-700 K. In Fig. 6, SVF simulates for R_c = 5.85 nm and R_c = 2.45 nm, and for temperature varied from 20-700 K, while the Goworek simulation (the red solid line) uses simulated data of Goworek et al.[21]. In the calculations of SVF for different temperatures, the change of the parameter q with temperature, which is simply calculated as, q(T) = q[η_0/η(T)], has been taken into account, where, q = 0.03 nm, and η_0 = 0.12.

Comparison of $E_{av}(\tau)$ used in SVF and that used by Nagashima *et al.*[3].

$E_{av}(\tau)$ defined by SVF is presented in the dependence of the pore radius, which is not the same with that defined by Nagashima *et al.*[3], which is in the time dependence. However, because the o-Ps lifetime depends on the pore radius as described by Eq. (23), one can simulate the $E_{av}(\tau)$ in the o-Ps lifetime dependence. In Fig. S4, the simulation of $E_{av}(\tau)$ with E_0 being 0.96 eV and μ_1 = 2.5 nm^{-1}/3, and the simulation of $\varepsilon_{av}^{(1)}(\tau)$ with the value of parameter, b_N = 1.3× 10^7 s^{-1} and with E_0 being 0.96 eV, are plotted against o-Ps lifetime. As shown in Fig. S4, $E_{av}(\tau)$ and $\varepsilon_{av}^{(1)}(\tau)$, which is described in Eq. (10) of ref. 3, are almost approximated. It is shown that using the SVF calculation presented by Eq. (23), the o-Ps lifetime dependence of $E_{av}(\tau)$ is consistent with time dependence of $\varepsilon_{av}^{(1)}(\tau)$ introduced by Nagashima et al.[3]. This result further supports the validity of the approach and expression of SVF.

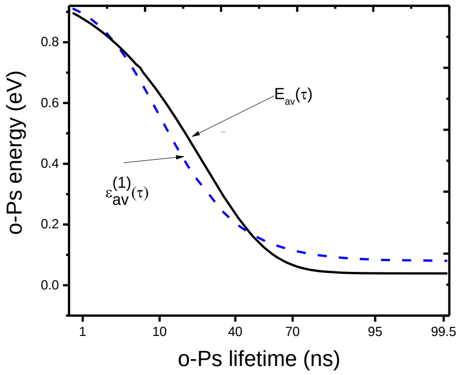

Fig. S4. The simulations of $E_{av}(\tau)$ used in SVF, and $\varepsilon_{av}^{(1)}(\tau)$ introduced by Nagashima et al.[3] are plotted against o-Ps lifetime (the solid line is simulation of $E_{av}(\tau)$ and dash line is simulation of $\varepsilon_{av}^{(1)}(\tau)$.

The temperature dependence of $E_{av}(\tau)$

The change of the energy of o-Ps annihilation, $E_{av}(\tau)$, due to the change of temperature from 20 to 700 K, is explained as follows. Consider to o-Ps being annihilation in given pore with effective pore radius, R_0. For different sample temperatures, $T_2 > T_0$ = 298 K. As presented above, the value of parameter, μ =2.5 nm^{-1}, as expressed in Eq. (5), is the value of $\mu(T)$ at T =298 K. The temperature change can cause the change of this value. Denoted $\mu(T_2)$ being the value of the parameter, $\mu(T)$, at temperature, T_2. The difference of the energies of o-Ps annihilation, $E_{av}(\tau)$, calculated for different temperatures, T_2 and T_0, is:

$$\Delta E_{av}(\tau) = E_0\exp[-\mu(T_2)R_0/3] - E_0\exp[-\mu R_0/3] + (3/2)k_B\Delta T_{Ps} \quad (26)$$

where $\Delta T_{Ps} = T_{Ps2} - T_{Ps0}$, T_{Ps2} and T_{Ps0} are temperatures of o-Ps gained from sample temperatures, T_2 and T_0, respectively. On other hands, the temperature change can cause the change of o-Ps lifetime[19-21]. The lifetime of o-Ps, τ_2, determined at temperature, $T_0 < T_2 \leq 700$ K, is smaller than that determined at T_0, $\tau_2 < \tau_0$,[5,10]. Therefore, $E_{av}(\tau_0) < E_{av}(\tau_2)$. According to the definition of $E_{av}(\tau)$, the change of the energy of the o-Ps, which is directly calculated by the temperature change as presented in Eq. (26,), can be indirectly calculated by the change of the o-Ps lifetime due to the change of temperature:

$$\Delta E_{av}(\tau) = E_{av}(\tau_2) - E_{av}(\tau_0) \quad (27)$$

Instead of using different values of $\mu(T_2)$ and μ in using the same value of R_0 as in Eq. (26), one can calculate $\Delta E_{av}(\tau)$ by using the same value of μ and different values of R_0 and R_{02} that SVF provide the values of τ_0 and τ_2. Supposed the value, R_{02}, gives the o-Ps lifetime of t_2, the change of annihilated energy of o-Ps:

$$\Delta E_{av}(\tau) = E_0\exp[-\mu R_{02}/3] - E_0\exp[-\mu R_0/3] \quad (28)$$

As presented in main text, the experimental and RTE calculation results for high temperature (298 -700 K) show thar τ_1/τ_2 <1.4, One can calculate the values of R_{02} giving the values, $\tau_2 = \tau_1/1.4$, by using SVF for τ_1 varying from 2 ns to 142 ns. The calculated result shows that:

$$\Delta E_{av}(\tau) = E_{av}(\tau_2) - E_{av}(\tau_0) < (7/2)k_B (T_1-T_2) = (7/2)k_B\Delta T \quad (29)$$

Eq. (28) gives:

$$E_{av}(\tau_2) - E_{av}(\tau_0) = E_0\exp[-\mu(T_2)R_0/3] - E_0\exp[-\mu R_0/3] + (3/2)k_B\Delta T_{Ps} \quad (30)$$

$$E_0\exp[-\mu(T_2)R_0/3] - E_0\exp[-\mu R_0/3] < 3k_B\Delta T - (3/2)k_B\Delta T_{Ps} = 2k_B\Delta T + (3/2)k_B(\Delta T - \Delta T_{Ps}) \quad (31)$$

For $\mu R_0/3 \leq 3$ or $R_0 \leq 3.6$ nm, expanding (31) to Maclurin series, one has:

$$\exp[-\mu(T_2)R_0/3] - \exp[-\mu R_0/3] \geq [\mu - \mu(T_2)]R_0/3 \quad (32)$$

If $k_B(\Delta T - \Delta T_{Ps}) \approx 0$, in the temperature range of 298-700 K, inequality (31) gives:

$$[\mu - \mu(T_2)]R_0/3 \leq \exp[-\mu(T_2)R_0/3] - \exp[-\mu R_0/3] < 0.07/E_0 \quad (33)$$

For 1 nm < R_0 < 3.6 nm, inequalities (31) & (32) give:

$$[\mu - \mu(T_2)]/\mu < 0.22\ nm^{-1}/2.5\ nm^{-1} < 0.088 \quad (34)$$

If (ΔT- ΔT_{Ps}) < 100 K, [μ - $\mu(T_2)$]/ μ < 0.095. This can make the relative change of o-Ps lifetime being less than 2% for R_0 > 1 nm.

For the cases, R_0 > 3.6 nm, consider:

$$\Delta E_{av}(\tau) = E_0 \exp\left[-\frac{\mu(T_2)R_0}{3}\right] - E_0 \exp\left[-\frac{\mu R_0}{3}\right] + \frac{3}{2}k_B \Delta T_{Ps} \le (7/2)k_B\Delta T \quad (35)$$

$$\exp\left[-\frac{\mu(T_2)R_0}{3}\right] - \exp\left[-\frac{\mu R_0}{3}\right] \le \frac{3(\Delta T - \Delta T_{Ps})}{2E_0} + \frac{2}{E_0}k_B\Delta T \quad (36)$$

If $k_B(\Delta T - \Delta T_{Ps}) \approx 0$, and R_0 > 3.6 nm, for temperature, 298 K < T ≤ 700 K, inequality (36) gives:

$$\exp\left[-\frac{\mu(T_2)R_0}{3}\right] \le \frac{2}{E_0}k_B\Delta T + \exp\left[-\frac{\mu R_0}{3}\right] \le 0.117 \quad (37)$$

From (37), one has: 1.787 $nm^{-1} \le \mu(T_2) \le \mu$ = 2.5 nm^{-1}, and the relative deviation, $[\mu - \mu(T_2)]/\mu \le 0.285$. If 0 < ($\Delta$T- ΔT_{Ps}) < 100 K, [μ - $\mu(T_2)$]/ μ < 0.312. For this value of the relative change of $\mu(T_2)$, and for R_0 > 3.6 nm, the relative change of the o-Ps lifetime being less than 2%. It is similar results can be given for the case, $T_0 > T_2 \ge 20$ K.

Therefore, one can use the room temperature value, μ =2. 5 nm^{-1}, to calculate the o-Ps lifetime for different temperatures, T = 20-700 K with the relative errors less than less than 2% for pore radius greater than 1 nm.

For R_0 < 1 nm, it is necessary to take into account the change of parameter, μ, to calculate the o-Ps lifetime of sample temperature, T, varied over range of 20 -700 K.

References

1. Gidley, D.W., Peng, H.G., & Vallery, R.S. Positron annihilation as a method to characterize porous materials. *Annu. Rev. Mater*. Res. **36**, 49-79 (2006).
2. Nagashima, Y., Morinaka, Y., Kurihara, T., Nagai, Y., Hyodo, T., Shidara, T. & Nakahara, K. Origins of positronium emitted from SiO_2. *Phys. Rev. B.* **58**, 12676 (1998).
3. Nagashima, Y., Kakimoto, M., Hyodo, T., Fujiwara, K., Ichimura, A., Chang, T., Deng, J., Suzuki, K., B. T. A. McKee, B. T. A. & Stewart, A. T. Thermalization of free positronium atoms by collisions with silica-powder grains, aerogel grains, and gas molecules. *Phys. Rev. A*. **52**, 258 (1995).
4. Shu, K; Ishida, A; Namba, T; Asai, S; Oshima, N; O'Rourke, B. E and Ito, K. Observation of ortho-positronium thermalization in silica aerogel at cryogenic temperatures. *Phys. Rev. A* 104, L050801 (2021).
5. Gidley, D. W., Frieze, W. E., Dull, T. L., Yee, A. F., Ryan, E. T. & Ho, H. M. Positronium annihilation in mesoporous thin films. *Phys. Rev. B*, **60**, R5157-R1560 (1999).
6. Tanzi, G. M., Castelli, F. & Consolati G. Positronium Confinement in Small Cavities: A Two-Particle Model for the Lowering of Contact Density. *Phys. Rev. Lett*. **116**, 033401 (2016).
7. Landau, L. D. and Lifshitz, E. M. *Quantum Mechanics*, Vol. 3, Pergamon, New York (1965).
8. Tao, S. J. Positronium Annihilation in Molecular Substances. *J. Chem phys*. **56**, 5499 -5510 (1972).
9. Alerhand, O. I., Joannopoulos, J. D. and Mele, E. J. Thermal amplitudes of surface atoms on Si(111)2 × 1 and Si(001) 2 × 1. *Phys. Rev. B*, **39**, 12622 (1989).
10. Dutta, D., Ganguly, B., Chatterjee, B & Mukherjee, T. Effect of Temperature on Positronium Annihilation in Silica Gel. *J. Phys. Chem. B*, **109**, 10092-10095 (2005).
11. Charlton, M. & Humberston, J. M. Positron Physics, *Cambridge University Press, Cambridge* (2000).
12. Consolati, G., Mariani, M., Millini, R. & Quasso, F. Investigation on the porosity of zeolite NU-88 by means of positron annihilation lifetime spectroscopy. *Nucl. Instrum. Methods. Phys. Res. B*. **267**, 2550-2553 (2009).
13. Puska, J. and Nieminen, R. M. Theory of positrons in solids and on solid surfaces. *Rev. Mod. Phys*. **66**, 841 (1994).
14. Dull, L., Frieze, W. E., & Gidley, D. W. Determination of pore size in mesoporous thin films from the annihilation lifetime of positronium. *J. Phys. Chem. B*, **105**, 4657-4662 (2001).
15. Eldrup, M., Lightbody, D & Sherwood, J. N. The temperature dependence of positron lifetimes in pivalic acid. *Chem. Phys*. **63**, 51-58 (1981).
16. Boronski, E and Nieminen, R. M. Electron-positron density-functional theory. *Phys. Rev. B* **34**, 3820 -3829 (1986).
17. Dutta, D., Feldblyum, J.I., Gidley, D.W., Imirzian, J., Liu, M., Matzger, A. J., Vallery, R.S., & A. G. Wong-Foy, A.G. Evidence of Positronium Bloch States in Porous Crystals of Zn4O-Coordination Polymers. *Phys. Rev. Lett*. **110**, 197403 (2013).
18. Crivelli, P., Cooke, D., Barbiellini, B., Brown, B. L., Feldblyum, J. I., Guo, P., Gidley, D. W., Gerchow, L. & Matzger, A. Positronium emission spectra from self-assembled metal-organic frameworks. J. *Phys. Rev. B*, **89**, 241103(R) (2014).
19. Dutta, D., Chatterjee, S., Pillai, K. T., Pujari, P. K., Ganguly, B. N. Pore structure of silica gel: a comparative study through BET and PALS. *Chem. Phys.* **312**, 319–324 (2005).
20. Ito, K., Nakanishi, H. & Ujihira, Y. Extension of the Equation for the annihilation lifetime of *ortho*-positronium at a cavity larger than 1 nm in Radius. *J. Phys. Chem*. B **103**, 4555-4558 (1999).
21. Goworek, T., Ciesielski, K., Jasin´ska, B., & Wawryszczuk, J. Positronium states in the pores of silica gel. *Chem. Phys.* **230**, 05-314 (1998).
